# Welfare at Multiple Scales: Importance of Zoo Elephant Population Welfare in a World of Declining Wild Populations

**DOI:** 10.1371/journal.pone.0158701

**Published:** 2016-07-14

**Authors:** Elissa Z. Cameron, Sadie J. Ryan

**Affiliations:** 1 School of Biological Sciences, University of Canterbury, Christchurch, New Zealand; 2 School of Biological Sciences, University of Tasmania, Hobart, Tasmania, Australia; 3 Department of Geography, University of Florida, Gainesville, Florida, United States of America; University of KwaZulu-Natal, SOUTH AFRICA

In-situ elephant populations have been in decline for much of the last 200 years, driven by an inexorable combination of habitat loss and hunting for ivory, but with more recent and dramatic declines primarily driven by hunting [1]. Consequently, the distribution and sustainability of elephant populations are now better predicted by human factors than ecological ones [2], underscoring the importance of societal factors in the ongoing survival of elephants. Despite growing awareness of the conservation crisis, hunting pressure has not abated. Instead, there has been a recent surge in harvest rates [3,4], more than doubling of harvest since 2007 [5]. The rates are staggering, escalating from an estimated 40,000 African elephants killed in 2011 [4] and 41 tons of ivory seized, to possibly more than 10% of the remaining populations in 2013 (summarised in [6]). In April 2016, the Kenyan Wildlife Service burned the biggest stockpile of ivory since it began burning ivory in 1989, with 105 tonnes of ivory destroyed, representing 6000–7000 poached elephants [7,8]. Similar declines have been seen in forest [3,9] and Asian [10] elephants. The current harvest rate of elephants is unsustainable, creating a conservation crisis of global significance, with an immediate threat to their continued survival [11]. Novel genetic tracing techniques (e.g [6]) and strict anti-poaching law enforcement [5] are vital to conserve the remaining free-ranging elephant populations.

The significance of the current overharvesting crisis extends beyond obvious direct impacts on the number of elephants, to their demography, through a variety of influences. As large bodied mammals, with complex social interactions, social structures, and extended weaning periods, the impact of disruptions to demography are long-lasting. For example, matriarchs are a repository of social information [12], such that their loss can have a disproportionate impact on social cohesion, herd demography and fitness, and these effects can last for decades [13]. Furthermore, physiological stress can be increased in areas where elephants are exposed to anthropogenic stress [14,15], with potential impacts on reproductive senescence and lifetime reproductive success [16]. Finally, overharvesting can influence ecological dynamics beyond the harvested species, affecting larger food webs and ecological processes in the landscape [4,17]—including the loss of a large-scale ecological engineer and important seed-disperser [18–21]. The disruption caused by poaching is likely to exacerbate issues of human-wildlife conflict involving elephants, affecting the lives and livelihoods of local human communities [22–24]. This can also lead to the vicious cycle of ‘retaliatory’ killing, compounding population impacts. Therefore, the conservation crisis extends beyond the animals killed by poaching, to broad ecosystem and community impacts.

Populations experiencing dramatic declines in numbers become susceptible to a variety of demographic influences not seen in larger populations, and result in susceptibility to an extinction vortex. Many ‘wild’ populations of elephants are now clearly in the ‘small population’ biology paradigm, in terms of conservation [25–27]. In addition, due to their extremely complex social system, large body size, and concomitant slow demographic turnover, plus potential for cultural isolation issues [28], understanding these influences is integral to managing for their survival. Consequently, all remnant populations of elephants become of vital importance to their continued existence, and can essentially be regarded as a metapopulation. Currently, only a minority of free-ranging elephants exist in large undisturbed protected areas [29]. Intensively managed populations in small reserves therefore become increasingly important for conservation, particularly since land tenure and type of land use impact conservation outcomes for elephants [30].

Intensively managed populations of elephants in small reserves closely resemble populations of elephants in zoo populations, some of which are kept in extensive enclosures, similar to small fenced reserves, and are important for future survival of the species. Valuable research conclusions can be based on non-wild populations, including both domestic and zoo animals [16,31], and in particular, factors that influence longevity, health and reproduction in zoo populations are likely to also play a role in wild populations, particularly those that are intensively managed. Furthermore, such information will be of particular value for future intensive conservation efforts, including translocations, reintroductions and possible rewilding (e.g. [32,33]). Thus, we call attention to the role of zoo elephant population research to in-situ conservation efforts, and stress the importance of understanding welfare, as a component of the scales of integral research into elephant conservation.

This collection of papers takes an epidemiological approach to exploring the connections between the daily lives of elephants in captivity and their welfare (introduced in [34]). By taking a multi-institutional approach, the collection is able to explore the overall trends in data without the usual small sample size issues that plague studies of animals in captivity [35]. As pointed out, the group of studies revealed that social and management factors were strongly implicated for multiple welfare indicators, while exhibit space was found to be less important [34]. The collection is timely, not only because of the broad public interest in captive animal care and management, as the authors themselves stress [34], but because amidst the global decline in elephants, these analyses have implications for conservation of a species in crisis, since welfare impacts demography, including longevity and reproductive success. Thus, effective research in captive facilities can fill key knowledge gaps, to which the series of papers contribute. By using a cross-institutional design, the authors are able to assess both institution-specific factors as well as factors that apply regardless of local management. In so doing, they assess factors associated with welfare, including housing and social environments [36], environmental enrichment [37], and social demography [38]. In addition, the authors assess impacts on physiological [39,40], physical [41,42] and behavioural outcomes [43,44], tying together patterns at multiple scales and dimensions.

This collection represents a timely collation of research using a collaborative, multi-institutional approach. This set of papers also represents the largest collected set of publications on the welfare of zoo elephants to date. Such a collaborative and innovative approach exemplifies the effort and research required to ensure the persistence of biodiversity globally, and elephants in particular.
